# Hormesis: Path and Progression to Significance

**DOI:** 10.3390/ijms19102871

**Published:** 2018-09-21

**Authors:** Edward J. Calabrese

**Affiliations:** Toxicology, University of Massachusetts, Environmental Health Sciences, Morrill I-N344, Amherst, MA 01003, USA; edwardc@schoolph.umass.edu; Tel.: +1-413-545-3164

**Keywords:** hormesis, dose response, adaptive response, pre-conditioning, biphasic, resilience, hormetic

## Abstract

This paper tells the story of how hormesis became recognized as a fundamental concept in biology, affecting toxicology, microbiology, medicine, public health, agriculture, and all areas related to enhancing biological performance. This paper assesses how hormesis enhances resilience to normal aging and protects against a broad spectrum of neurodegenerative, cardiovascular, and other diseases, as well as trauma and other threats to health and well-being. This paper also explains the application of hormesis to several neurodegenerative diseases such as Parkinson’s and Huntington’s disease, macrophage polarization and its systematic adaptive protections, and the role of hormesis in enhancing stem cell functioning and medical applications.

## 1. Introduction

The dose response concept is central to biology, medicine, and public health [1]. It represents the biological integration of how living systems at all levels of organization, from the cell to the individual, respond, adapt or fail to adapt to endogenous agents, metabolic processes, and externally imposed stressors/threats. The dose response can capture and provide biological/mechanistic insight to such challenges when assessed as a dose-time response that describes dynamic processes such as the induction of toxicity, repair, and recovery [2]. Biological systems are therefore dynamic entities with an evolutionary adaptive strategy, which is reflected in the nature of the dose-time response.

## 2. Dose Response in Historical Context

Within this context consider how the radiation genetics research community approached the concept of dose response for radiation-induced mutation which was discovered by Muller [3]. Within three years of this discovery Muller [4] proposed the so-called “Proportionality Rule”, that the dose response for X-ray induced mutation was linear down to a single ionization for all cell types [5,6]. This perspective would subsequently lead to the creation of the linear non-threshold (LNT) model for low dose risk estimation [7]. The Proportionality Rule was widely viewed as credible by the contemporary radiation genetics community and eventually would be accepted by the US National Academy of Sciences (NAS) Biological Effects of Atomic Radiation (BEAR) Genetics Panel [8], whose recommendations lead to the adoption of the LNT model for cancer risk assessment worldwide. The Proportionality Rule and its direct regulatory dose response progeny, the LNT cancer risk model, were products of a series of three key assumptions that all induced genetic damage was: (1) unrepairable; (2) irreversible; and (3) cumulative. This collective and integrative set of functional assumptions lead to the belief that the dose response would be linear for ionizing radiation and chemical-induced mutation and carcinogenesis.

This dose response hypothesis was based on the study of mutations in mature spermatozoa of Drosophila. At the time of this hypothesis formation and its applications to risk assessment, it was not known that DNA repair existed let alone that it became lost or fully degraded as spermatogonia transitioned to mature spermatozoa. Thus, when the US NAS Genetics Panel [8] made their seminal recommendation that regulatory agencies adopt the LNT model for risk assessment they were fully committed to the belief that X-rays and chemical mutagens would cause mutations that could never be repaired and that any induced damage would be cumulative and the dose response linear. So firm were these beliefs within the radiation genetics community that these incorrect convictions quickly became public policy, remaining so even today, as reflected in national regulatory risk assessment policies.

This belief in linearity at low doses would be challenged by another contemporary prominent radiation geneticist, William Russell, Oak Ridge National Laboratories, using the mouse specific locus test with over two million mice in his iterative radiation-induced mutational studies (see [9,10] for a detailed review). In this case, Russell [11,12] employed mouse spermatogonia (rather than the mature spermatozoa) as the stage of reproductive cell exposure. Russell discovered the concept of dose rate, that is, the mutation damage was not cumulative but a function of the rate at which the radiation was applied. This finding was directly contradictory to the longstanding geneticist mantra of irreversible, cumulative, and linear. Based on his dose rate studies Russell [13] reported that female mice, even when administered a radiation dose some 27,000 times greater than background ionizing radiation, displayed a mutation response that was not greater than the mutation rate of the unexposed controls. This dose-time experiment would reveal that the oocytes were not static cells but able to prevent/repair fully the effects of even relatively high doses of radiation. These observations as well as those with spermatogonia lead Russell to propose the existence of DNA repair processes. Within the next four year’s such DNA repair processes were shown to exist, be very general and eventually lead to the discovery of constitutive and inducible repair processes and to an eventual Nobel Prize in 2015. These findings would also lead to the recognition that mature spermatozoa lack such DNA repair but that this deficit is compensated by repair capacities provided by the ovum during the reproductive process.

The Russell et al. [12] dose rate discovery was a seminal event and led to the recognition that the US NAS BEAR Genetics Panel [8] made an error in their extrapolation of responses in mature spermatozoa to all cells, including somatic cells. These findings of Russell revealed that the linear dose response mantra of the radiation geneticists was no longer valid [14,15]. This BEAR Genetics Panel error [8], which was recognized by the next US NAS BEIR (Biological Effects of Ionizing Radiation) Committee [16], was of fundamental importance as it incorrectly provided the scientific foundation for the linear dose response for mutation and cancer risk assessment. Not recognized by the BEIR Committees (1972 to the most recent 2006) was that Muller’s assertion that he had produced gene mutations in his 1927 published research was also incorrect. Based on modern nucleotide analyses Muller’s mutations were not gene mutations but genetic alterations at the level of chromosome due principally to modest to massive gene deletions [17]. The LNT model was therefore derived based on the incorrect assumption that Muller had induced gene mutations. Either of these errors (i.e., extrapolating from mature spermatozoa to somatic cells and the gene mutation interpretation mistake) would invalidate the LNT model.

The Russell findings would also provide the foundation for subsequent research which demonstrated the existence of the adaptive/repair responses for chemically induced mutations in the mid-1970s [18] and later for ionizing radiation [19]. These adaptive response/preconditioning protective findings were significant not only because they affected mutation production, which was considered the underlying mechanism of cancer, but because they paved the way for extending the preconditioning concept to a vast range of other diseases and injuries that could be prevented/minimized by hormetic strategies [9,10].

The existence of a broad spectrum of DNA repair and other adaptive processes lead to the belief that the linear dose response was at best extremely limited, with little or no application to complex biological systems [20]. The discovery of various classes of protective mechanisms also led to new dose response models or giving old dose response models a second look. Within the framework of ionizing radiation exposures were the dramatic findings of Azzam et al. [21] that very low doses of radiation had the capacity to upregulate DNA repair processes leading to a significantly lower amount of mutational damage than found in the normal controls, thereby suggesting the existence of an hormetic-biphasic dose response. Furthermore, numerous studies have shown that removal of background radiation from the environment of various experimental models leads to striking degradations of health status, which is reversible with the reinstituting of the background radiation [22,23].

## 3. The Hormesis Concept

This brief historical vignette into the foundations of the radiation genetics-mutation story demonstrates that this research community had a concept that biological systems, from cells to whole organisms, were static rather than dynamic. It was also curious that these evolutionarily-based radiation geneticist researchers would not have assumed that selection for genetic repair processes would have occurred during evolution and be reflected in the mutation dose response. Of particular note is that the developments of Russell, Samson and Cairns [18], Olivieri et al. [19], and Azzam et al. [21] suggested that dose responses in the low dose zone were not only not linear but possibly not even a threshold but probably biphasic (i.e., J-shaped or inverted U-shaped depending on the endpoint graphed). These findings were supported subsequently by massive summaries of ionizing radiation dose response information provided in seminal books by Luckey [24,25]. These books supported the premise that the hormetic dose response was so common in the scientific literature that hormesis should have been an evolutionary expectation rather than an exception, methodological error, or a response confused with background variation.

The gradual unfolding of the widespread nature of the hormetic dose response in the radiation genetics community would also occur in other areas of the biological and biomedical sciences from the late 1970s to the present. These researchers developed new analytic tools to measure progressively lower doses of chemical agents and exploited the in vitro revolution of the 1980s which permitted the testing of a large number of concentrations across broad concentration ranges. These developments are reflected in the number of citations with the term hormesis or hormetic on a yearly basis in the Web of Science data base. During the entire decade of the 1980s this number was about 10–12 citations per year whereas in 2017 alone this number increased dramatically to over 9300, reflecting a highly interdisciplinary reporting of hormetic dose responses with a strong mechanistic framework.

## 4. How Medicine, Pharmacology, and Toxicology Got the Dose Response Half-Wrong

While the hormesis concept was markedly accelerated by the above developments in cellular models and evaluation methods, the concept of hormesis is about 130 years old, starting with the findings of Hugo Schulz [26,27] concerning the effects of nearly a dozen disinfectants on yeast metabolism. Schulz’s research was significant as it established the biphasic dose response within the framework of a modest overcompensation response to an initial disruption in homeostasis. This research revealed the capacity of the organism to display an initial harmful response to toxic agents and to respond to such-induced damage with a modest reparative overcompensation (i.e., a low dose stimulation). Despite the fact that Schulz’s work was replicated by multiple groups [28] and broadly extended and generalized [29,30,31,32,33], Schulz made a fundamental error soon after his discovery by associating his biphasic dose response with the practice of homeopathy, asserting that he had discovered its explanatory principle [34]. This action of Schulz created a prolonged scientific and professional/personal backlash from the powerful traditional medical community curtailing his career and profoundly blunting acceptance of the biphasic dose response/hormesis concept [34,35,36].

Even though Schulz and his biphasic dose response (which he termed the Arndt-Schulz Law) would be stridently and unrelentingly challenged and ridiculed by leaders within traditional medicine [37,38,39], many independent investigators during Schulz’s professional career (1880 to 1932) would report similar biphasic dose response findings employing various biological models, especially in the fields of microbiology, plant biology, and entomology, using numerous chemicals and ionizing radiation [29,30,31,32,33]. However, for a variety of reasons (Calabrese [5,36]) the biphasic dose response concept continued to be marginalized, never integrated within the scientific mainstream. This would eventually begin to change in the 1970s as a result of the scientific leadership of Luckey [24], University of Missouri, with respect to ionizing radiation, Tony Stebbing [40,41], Plymouth Marine Research Station (UK), who studied heavy metal marine toxicology and Elmer Szabadi [42], University of Liverpool, who reported a large number of pharmacologically-based biphasic dose response relationships, placing them within a receptor based mechanistic framework. These three independent investigators provided the key intellectual foundations for the hormetic biphasic dose response transformation, which was sparked by the first hormesis conference that was held in Oakland, California in August, 1985 (with the peer reviewed proceedings published in the journal Health Physics in 1987).

The next several decades would provide substantial documentation that hormetic dose responses were common, and occurred in a broad range of microbial, plant, and animal models, suggesting widespread generality [43,44,45]. Likewise, the occurrence of hormetic responses could be induced by a wide range of agents, affecting a similarly wide range of endpoints [46,47,48]. These efforts led to the development of a continuously expanded hormesis data base that includes information on approximately 40 study related experimental and dose response features.

Despite the considerable diversity of biological models, inducing agents, and endpoints showing hormetic dose responses, they have one highly consistent characteristic in common, that is, their quantitative features are similar. This was also the case whether the low dose stimulation occurred via a direct stimulation or via an overcompensation to an initial toxicity response [49]. The stimulatory response magnitude at maximum was typically modest, with a maximum range of about 30–60% greater than the control group. This finding was reaffirmed as the number of hormetic studies increased from hundreds to many thousands, it being the most predictable feature of hormesis. This modest stimulatory response was independent of biological model, level of biological organization, endpoint and inducing agent. It was later shown to be independent of mechanism [50]. This further suggested that hormesis was providing a quantitative description of a form of biological plasticity [51]. 

These biologically profound developments occurred via the study of vast numbers of dose responses and their quantitative, and mechanistic features. Furthermore, the quantitative features of the hormetic dose response also were biologically modeled and reflected an allometric pattern that was similar to those that relate a vast range of key biological parameters to body weight and/or body surface [6]. These collective developments indicated that hormesis is a fundamental principle in biology, the product of natural selection and highly conserved. This biological principle had been overlooked by multiple generations of biological and biomedical scientists due, in large part, to the prolonged conflict between homeopathy and traditional medicine, the exclusion of the hormesis concept from mainstream scientific activities, and inherent challenges in studying modest responses at relatively low doses [34]. These key features, understandings and insights have been added since the mid-1990s as a result of the long term consistent focus on hormesis. 

Since hormesis defines the magnitude and limits of biological plasticity it has widespread and significant implications for drug development, disease resistance and other activities designed to enhance biological performance and to acquire resilience via procedures such as pre-and post-conditioning [9,10]. Since the constraints of plasticity are defined by the quantitative features of the hormetic dose response this perspective can inform the pharmaceutical and agrochemical industries that biological performance is maximally limited to the 30–60% range, having important implications for study designs and commercial product exploration in the clinic, laboratory, and field studies.

The evaluation of the maximum hormetic stimulatory response represents a type of biological optima. Exactly where that optimal hormetic stimulation response resides in the zone below the threshold is important to determine in the testing of possible therapeutic agents. It is frequently located in a zone usually 10–20 fold below the estimated threshold. Using the hormesis data base [46,47] we have evaluated whether the number of doses below the threshold may affect the capacity to detect the optimal dose (i.e., dose predicting the maximum stimulatory response). This set of evaluations revealed that with one dose below the threshold the maximum observed stimulatory response was approximately 20% above the control response. As the number of doses below the threshold increased to 6–7 the maximum observed stimulatory response progressively increased to approximately 60% greater than the control value. Thus, the tendency of clinical trials to use one to two doses suggests that such studies will yield a lower maximum stimulation than biologically possible. This suggests the need for either a larger sample size to detect a significant treatment effect and/or additional treatment groups within the hormetic zone. This insight into how the number of number of doses below the threshold affects the maximum hormetic stimulation may become a factor for those interested in assessing hormetic doses response in all types of studies as this relationship occurred whether the studies were conducted in vitro or in vivo and independently of biological model and endpoint.

## 5. Hormetic Applications

The hormetic dose response has important implications for the fields of hazard assessment, risk assessment for carcinogens, endocrine disruption, for pharmaceuticals/natural products that enhance biological performance, and pre/post conditioning activities that upregulate adaptive mechanisms, enhancing resilience.

### 5.1. Hazard Assessment

Hormesis is helpful in guiding the hazard assessment process in several ways. It provides a scientific basis for the selection of biological models for evaluation especially as it relates to background disease incidence. It provides a dose response model framework to assist in the selection of the number of doses, the dose spacing, and sample sizes to be employed. Hormesis also affects decisions concerning repeat sampling over time to assess possible compensatory responses. These hormetic-based insights are useful and likely to enhance the quality and utility of such studies, increasing confidence in the findings while also providing biostatistical model validation. Nonetheless, these factors can be problematic since they make experiments more expensive and longer to complete. Furthermore, in order to be successful in locating doses below the threshold one must know where the threshold is likely to occur in order to better target the low dose hormetic stimulatory zone with appropriate study designs. This would also usually require additional preliminary experiments to better clarify the dose zone within which a threshold may be likely to occur. These experimental challenges can make it difficult to assess hormetic hypotheses and to provide reproducible findings for low dose responses. This can be troublesome if the control group is especially variable. Further, it is important to have well documented information on control group variation. Failure to take such factors into consideration has the potential to reduce confidence concerning whether observed hormetic-like biphasic dose responses are reproducible effects or simply due to background variation.

### 5.2. Risk Assessment for Carcinogens

**Figure 1:**
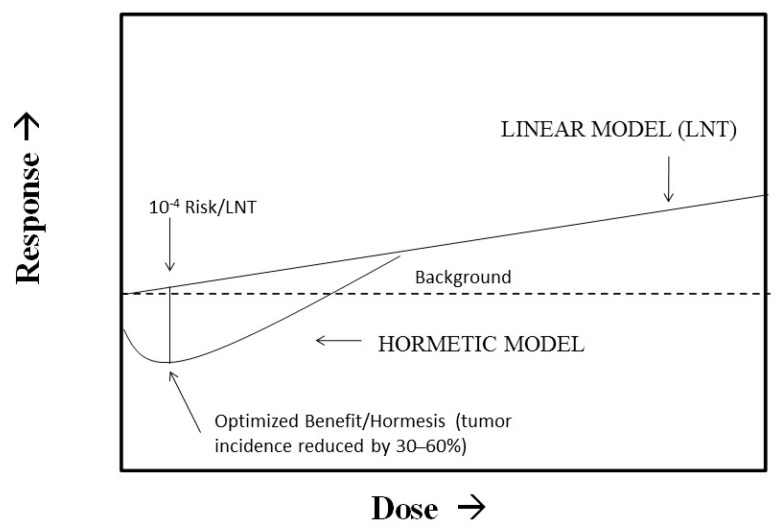
Integration of hormesis and LNT (linear non-threshold) for risk assessment. (Source: Calabrese et al. [52]).

It has recently been proposed that regulatory agencies such as the Environmental Protection Agency (EPA) move away from their use of the LNT as the default model in cancer risk assessment and consider the use of non-linear dose response models such as hormesis. Calabrese et al. [52] proposed that this EPA proposal could be implemented with a “model optimization” approach that integrates the best features of the LNT, threshold, and hormetic models. We have previously shown that using a Bench Mark Dose (BMD) plus a 100 fold uncertainty factor method as is typically used for a chronic toxicity threshold risk assessment method closely approximates the same dose at the nadir of the hormesis curve where health benefits are optimized [53]. This dose also represents an LNT risk of about 10^−4^ (Figure 1). This model uncertainty approach therefore identified a type of regulatory sweet spot wherein the population risk increases when the dose changes either up or down from the optimized dose at the nadir of the hormetic curve. In this approach, the LNT may be considered as the upper bound of uncertainty while the hormetic model would represent the lower bound. This approach also provides a type of dose convergence validation of the hormetic approach via the threshold model even though the risk interpretations differ at the optimized dose. If the hormetic model were correct, large health benefits would accrue to the population at risk. However, if the hormetic model were incorrect and the LNT were fully correct, the effects could not even be detected by the most powerful epidemiological studies. In fact, the increased risk would still be about 1/500 of the background cancer risk, thereby making this model uncertainty approach for cancer risk assessment practical and attractive from a public health perspective.

### 5.3. Harmful Effects of Hormesis

Low doses of some endocrine disrupting agents act via biphasic dose responses that conform very closely to the quantitative features of the hormetic dose response. For example, in some animal models low doses of bisphenol A (BPA) biphasically affected prostate size, with a maximum enhancement in the hormetic response zone [54]. A similar type of hormetic-like biphasic biological response occurs for many anti-tumor drugs when tested on dozens of human tumor cells lines [55]. Similar hormetic effects have also been reported for large numbers of antibiotics in broad screening assays [56]. There are numerous other types of examples where the low dose stimulation is considered an adverse effect [44]. The magnitude of these effects are also constrained by the bounds of biological plasticity.

### 5.4. Pharmaceutical Products

**Figure 2:**
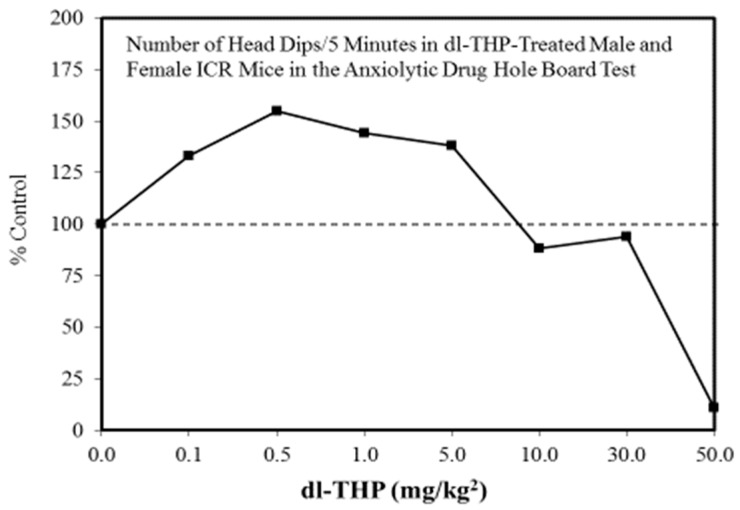
Number of head dips/5 min in (dl-THP)-treated male and female ICR mice in the anxiolytic drug hole board test (Source: Leung et al. [67]).

**Figure 3:**
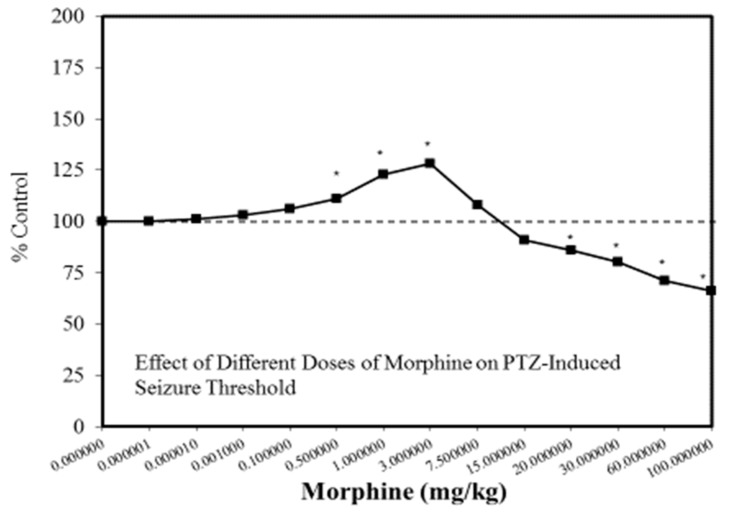
Effect of different doses of morphine on pentylenetetrazole (PTZ)-induced seizure threshold (Source: Honar et al. [68]).

**Figure 4:**
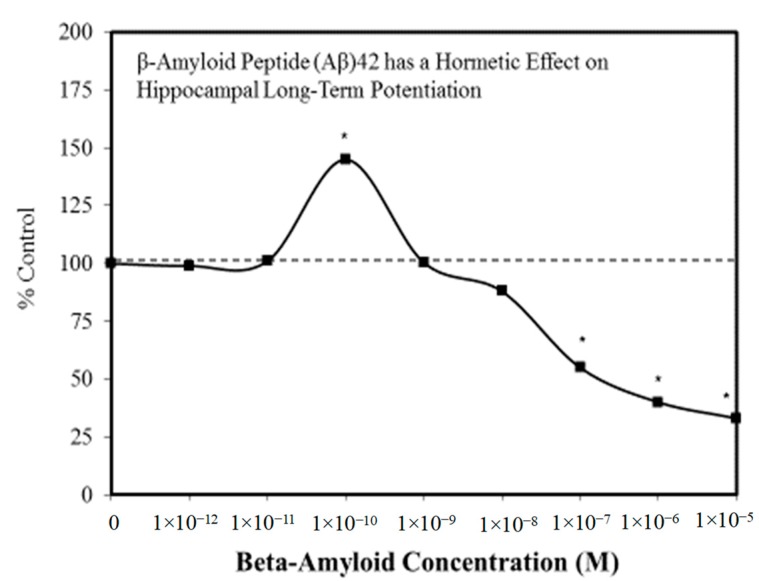
β-amyloid peptide (Aβ)42 has a hormetic effect on hippocampal long-term potentiation (Source: Puzzo et al. [59]).

**Figure 5:**
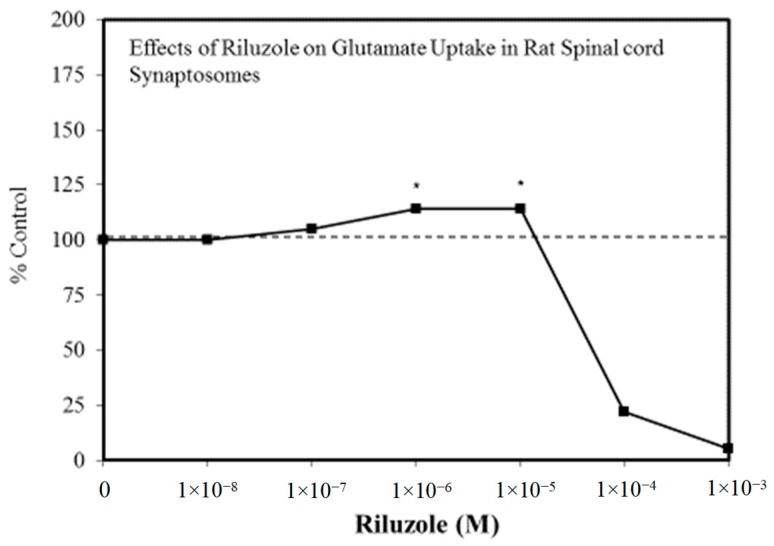
Effects of riluzole on glutamate uptake in rat spinal cord synaptosomes (Source: Frizzo et al. [60]).

A wide range of pharmaceutical products display hormetic features, including anxiolytic (Figure 2) [54] and anti-epileptic (Figure 3) [57] drugs. In preclinical studies, these agents uniformly exhibit hormetic dose responses. Based on these findings with animal models, the optimized dose would be selected for testing within human subjects. Thus, the pharmaceutical industry has substantial areas of its product portfolio based on the hormetic dose response. The area of memory enhancing drugs likewise displays the hormetic dose response [58]. All Alzheimer’s Disease (AD) drugs approved for human use by the US FDA (Food and Drug Administration) display the hormetic dose response in their preclinical findings [58]. While there has been intense research activity on how to prevent the accumulation of beta-amyloid plaques in the brains of AD patients, beta amyloid has essential biological functions and acts in a hormetic-biphasic fashion [59] (Figure 4), findings that have been emphasized in the AD research community but yet still remains little appreciated nor clinically exploited. Furthermore, the longest approved drug for the treatment of ALS (amyotrophic lateral sclerosis) is riluzole [60], which has its protective functions mediated via the induction of hormetic processes (Figure 5).

### 5.5. Parkinson’s Disease and Huntington’s Disease

**Figure 6:**
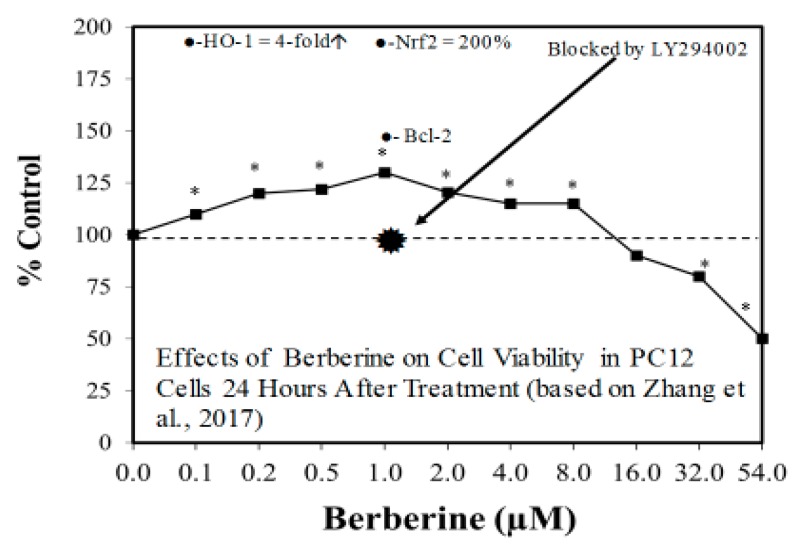
Effects of berberine on cell viability in PC12 cells (i.e., Parkinson’s disease cellular model) 24 h after treatment (Source: Zhang et al. [69]).

**Figure 7:**
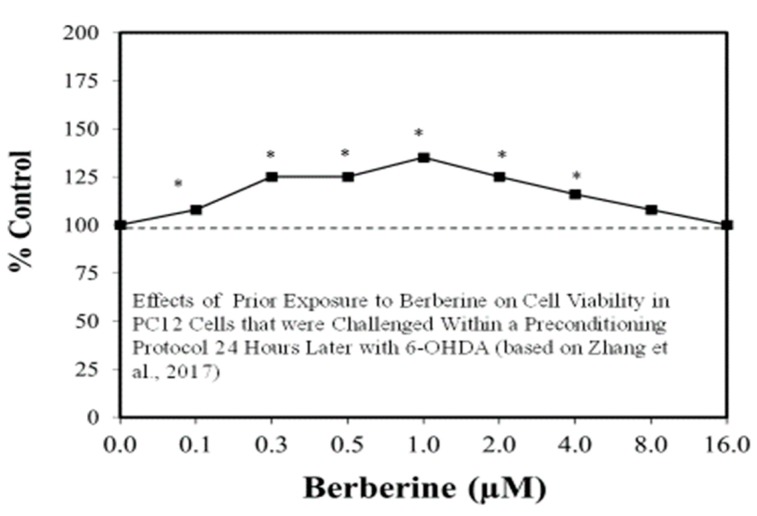
Effects of prior exposure to berberine on cell viability in PC12 cells that were challenged within a preconditioning protocol 24 h later 6-hydroxydopamine (6-OHDA) (Source: Zhang et al. [69]).

**Figure 8:**
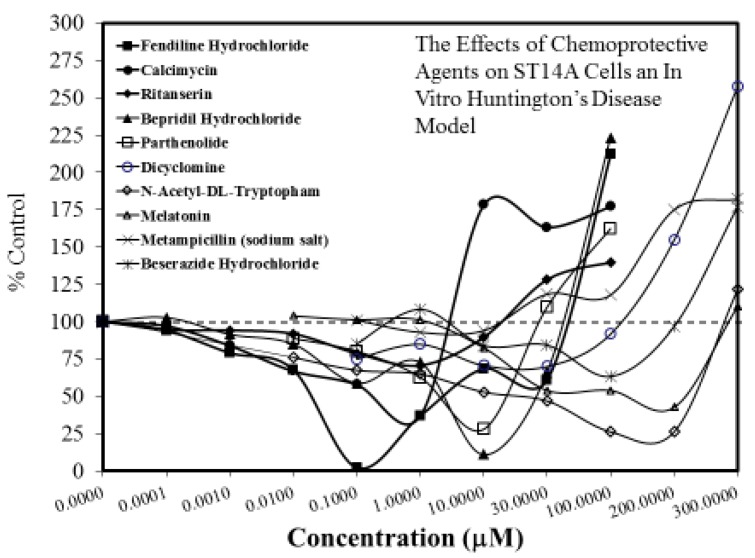
The effects of chemopreventive agents on ST14A cells an in vitro Huntington’s disease model (Source: Wang et al. [63]-Table S1). Protection occurs in responses <100%, whereas toxicity occurs for responses >100%.

**Figure 9:**
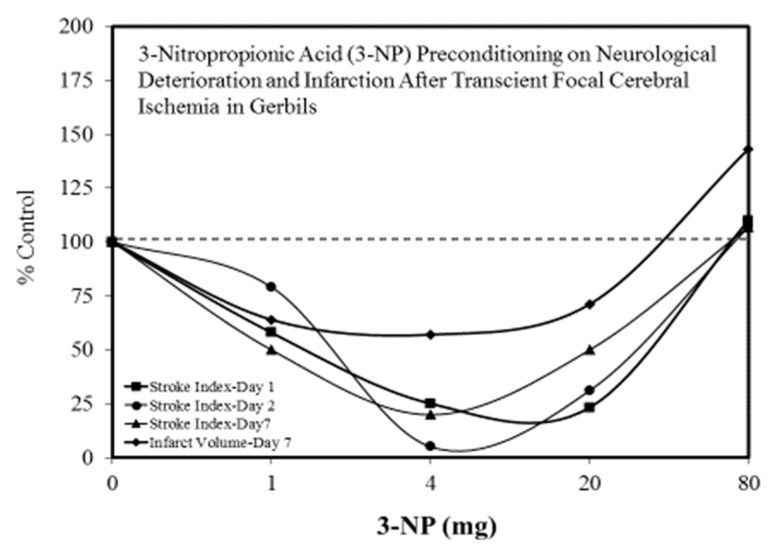
3-Nitropropionic acid (3-NP) preconditioning on neurological deterioration and infarction after transient focal cerebral ischemia in gerbils (Source: Kuroiwa et al. [68]).

The search for viable treatments for Parkinson’s and Huntington’s Diseases (HD) use a variety of predictive experimental models. Calabrese et al. [61,62] have reported the widespread occurrence of promising chemopreventive agents for Parkinson’s Disease (PD). In this assessment, approximately 50 agents were found to affect chemoprotection via hormetic processes (Figure 6 and Figure 7) [62]. The large number of hormetic examples of possible PD agents has been enhanced by the use of cellular model systems that incorporated relatively large numbers of concentrations, permitting more detailed evaluations of the dose response features in the therapeutic zone. This offers a novel effort to explore, evaluate and frame potential drug discovery and therapeutic applications within an hormetic framework. This has also been the case with HD with numerous examples of hormesis biphasic dose responses in in vitro (Figure 8) [63] and in vivo experimental models (Figure 9) [64]. In the case of both PD and HD the experimental protocols have made extensive use of hormetic-preconditioning protocols. Other experimental protocols have also demonstrated hormetic effects when the administration occurred at the initiation of the disease process or within post-conditioning frameworks. The capacity of the agents to activate hormetic processes within such a range of disease activating protocols is an important observation as it indicates that hormesis may be employed in prevention as well as therapeutically. Similar developments are also being reported within predictive models for other neurological diseases such as Multiple Sclerosis [65,66].

## 6. Hormetic Mechanisms

The role of mechanism in hormesis has shown marked advances over the past decade. During the 1990s as the concept of hormesis was beginning to be explored in depth, there were many reproducible examples of hormesis, but any mechanistic basis for such responses was very limited and often speculative. This has changed with major advances in the area of cell signaling pathways and their linkage with receptors activating biological processes. In 2013, Calabrese [50] documented mechanisms for 400 different hormetic dose response relationships at the level of receptor and cell signaling pathways. This assessment indicated that a vast range of mechanisms mediate hormetic responses. The quantitative features of the hormetic dose responses were independent of mechanism. This raises the question of why and how the quantitative features of hormesis are so similar across phyla, organs, cell types, endpoints, and proximate mechanisms within this context. Calabrese and Mattson [51] have identified possible regulatory biological processes emerging from allometric gene clusters. These genes regulate key biological traits such as the relationship of size of organs to overall body size. Hormetic dose responses also conform to allometric modelling and may be assessed within this biologically based allometric architectural gene cluster framework. These types of traits provide insights on overall form and function parameters from single celled organisms to humans and may offer clues to the basis for how the 30–60% increase in hormetic maximal responses occur that are independent of proximate mechanisms.

### Macrophage Polarization

A major development over the past two decades has been the recognition that macrophages can be reprogrammed toward pro-oxidative (called M1 macrophages) or anti-inflammatory forms (called M2 macrophages). In the case of preconditioning, this process acts to polarize macrophages toward the M2 form, facilitating protective/reparative/anti-inflammatory responses. We have recently hypothesized that macrophage reprogramming with polarization to M1 or M2 macrophage forms may be mediated via concentration gradients of signaling agents. A comprehensive assessment revealed many substances can mediate macrophage polarization following a concentration gradient that conforms to an hormetic dose response [70,71]. This concentration gradient regulatory strategy is widespread, affecting all organs. The initial incentive to explore this area was based on observations that low doses of radiation kills tumor cells while also ameliorating inflammatory conditions via the creation of anti-inflammatory phenotypes [72,73]. In the attempt to better understand the occurrence of radiation induced pro-oxidative and anti-inflammatory phenotypes, the challenge became greatly expanded as noted above. The integration of the hormesis concept within macrophage polarization reveals that inflammatory processes can be regulated as to facilitate the elimination of tumor cells and harmful microbes or conversely to suppress inflammatory processes and enhance healing via a concentration gradient process. These findings expand the biomedical/therapeutic significance of the hormetic concept within the context of biological regulatory processes and their biological/clinical applications.

## 7. Stem Cell Biology & Hormesis

A substantial literature exists on the occurrence of hormetic dose responses in a broad spectrum of stem cells. These stem cell hormetic responses have involved both direct stimulatory dose response features as well as those induced within a preconditioning framework. The quantitative features of the hormetic dose response for stem cells are similar to all other cell types [47]. Particular interest has focused on preconditioning-hormetic protocol with stem cells within a potentially therapeutic framework of tissue regeneration following damage from various conditions such as heart attack or stroke. The preconditioning hormetic process is expected to enhance the capacity of the injected stem cells to better survive very challenging biological micro-environments and enhance the likelihood of tissue repair [74,75,76,77,78]. An assessment of the Hormesis Database indicates that several dozen agents have induced hormesis in stem cells, with the most extensive research efforts with ionizing radiation, hypoxia, and resveratrol.

## 8. Discussion/Conclusions

This paper is set within the theme of this special journal issue—“day break hormesis”. Even though hormesis is scientifically “coming of age” it was first reported in the 1880s, with considerable research providing a large number of well executed studies through the past century in many biological areas [29,30,46,47]. Thus, one would have thought that the dawn of hormesis should not have been so drastically delayed, nor a fundamental biological principle missed by the scientific community for over a century. These bizarre historical circumstances would probably not have occurred had Hugo Schulz not associated hormesis with homeopathy and if homeopathy had not been in a major economic and intellectual war with what may be called traditional medicine [35]. While homeopathy was severely defeated in this real conflict, traditional medicine extended its hostility to its “explanatory principle”, the hormesis concept. Thus, via the leadership of key world leaders in the pharmacology community, such as Alfred J. Clark, homeopathy became nearly dismantled as a medical practice and Schulz would see a very fast rising career, quickly stalled and never to recover. In fact, in the US there were 23 homeopathic medicine schools in 1900 and only three about 20 years later [79,80]. As the fate of homeopathy was in a strongly downward spiral so to was its “explanatory principle”. Hormesis would not be found in any of the leading textbooks; it was excluded from research funding, and faculty at leading institutions were strongly discouraged from studying it. In effect, an idea was given a type of death sentence. This was as true academically as it has been in regulatory agencies worldwide. On top of this nearly impossible situation, the study of hormesis was also very challenging, requiring more doses, larger sample sizes, greater need for replication, and even more preliminary investigations to better identify the threshold zone. Superimposed on these problems was a dire lack of leadership and organization by those researching in this area. This has been investigated to a considerable depth and it was found that many of the leading hormesis researchers in the 1920s and 1930s moved to major institutional administrative positions, denying this troubled fledgling area the chance to grow [36]. What this amounted to was the failure of the scientific community to study, assess, and possibly recognize the significance of hormesis. In a great irony, this failure to grasp the hormesis concept occurred as a result of traditional medicine winning its battle with homeopathy. That is, traditional medicine won the war, but hurt its profession and the public with the discrediting of the hormesis concept. Making this situation even more problematic is that a vast number of terms can be used to describe the hormesis dose response. In fact, the very large hormesis data base [47] contains information from studies in which hormesis or hormetic were used as key words for only about 15% of the entered studies. Thus, hormetic dose responses are not easily found in the literature using the two key words: hormesis or hormetic.

The past century of challenges to hormesis has become less influential with its effects becoming diluted in an asymptotic-like regression. The past 30 years of intense research focus on hormesis has worked its way through this quagmire of historical antipathies and scientific challenges, with major advances establishing the generality of hormesis and its potential medical, public health, and agricultural implications.

Hormesis is now becoming prominent in leading textbooks in toxicology, pharmacology, and related biomedical areas. It has greatly profited from outstanding leadership in the areas of biogerontology, neuroscience/neurodegenerative diseases, exercise science, and other areas of public health interest. Hormesis is becoming seen as a central biological concept that can help researchers answer key biological questions in many domains. Thus, while it has been a 130 year wait, I am sure that Hugo Schulz would be proud that hormesis has finally reached its own scientific tipping point as reflected in this special issue on day break hormesis.

## Abbreviations

AD	Alzheimer’s Disease
ALS	Amyotrophic Lateral Sclerosis
BEAR	Biological Effects of Atomic Radiation
BEIR	Biological Effects of Ionizing Radiation
FDA	Food and Drug Administration
HD	Huntington’s Disease
LNT	Linear Non-Threshold
NAS	National Academy of Sciences
PD	Parkinson’s Disease
